# Correcting the Mean-Variance Dependency for Differential Variability Testing Using Single-Cell RNA Sequencing Data

**DOI:** 10.1016/j.cels.2019.08.003

**Published:** 2019-10-23

**Authors:** Nils Eling, Arianne C. Richard, Sylvia Richardson, John C. Marioni, Catalina A. Vallejos

## Main Text

(Cell Systems *7*, 284–294.e1–e12; September 26, 2018)

While revising code used to analyze the data described in Eling et al. (2018) for inclusion in a Bioconductor workflow associated to the BASiCS package, we discovered a problem with the analyses of datasets generated using the Fluidigm C1 system (Antolović et al., 2017, Lönnberg et al., 2017, Martinez-Jimenez et al., 2017). Specifically, we observed that the number of spike-in molecules present in the cell lysis volume had been miscomputed in our original analyses, such that all true spike-in numbers were inadvertently scaled by the same constant factor. Where available, these quantities are used as an input for BASiCS, and therefore, some of the outputs originally reported in Eling et al. were incorrect.

In principle, the original error in calculating the exact number of spike-in molecules per reaction only scales the arbitrary units in which gene expression is measured and thus should not alter any interpretation or downstream analysis. However, when we re-analyzed these data to correct Eling et al., we noted that mis-scaling led to a poor initialization of the MCMC sampler, which led to less stable estimates of the mean and dispersion for lowly expressed genes. In essence, more iterations were needed to achieve optimal good convergence. Consequently, using the correct input spike-in numbers leads to changes in the set of differentially expressed and variable genes identified.

In this Correction, we have re-generated all figures in our manuscript where the C1-derived datasets were used: Figures 1, 2, 5, 6, S5, and S6. In Figures 1 and 2, all panels depicting log (mean expression) have been replaced so that counts are given on the correct scale. In Figures 5, 6, S5, and S6, all panels have been changed so that data are given on the correct scale. In addition, some genes used as exemplars in Figures 5B, 6C, 6D, and S5 have been changed as those used previously fall slightly below the significance threshold. Figure legends have been updated accordingly. These changes are also reflected throughout the text as the different exemplar genes are discussed. Specifically, in Figure 5, where we considered changes in the mean and variability of gene expression levels during CD4 T cell activation, conclusions drawn about *Sf3a3*, *Plrg1*, and *Ncl* have been replaced with conclusions drawn about *Prpf31*, *Snrpa*, *Snrpd2*, *Larp1*, and *Eif3j2*; conclusions drawn about *Fasl* have been removed. When considering outlier cells that might drive expression heterogeneity, the example gene *Plrg1* was replaced with *Smad3*, and the example gene *Il2* from the simulated mixed population dataset was replaced with *Nfkbid*. Conclusions drawn about all other genes—*Polr2l*, *Polr1d*, *Naf1*, PD-L1 (*Cd274*), *Smad3*, *Pou2f2* and *Il2*—remain unchanged. In Figures 6C and 6D, conclusions drawn about example genes *Tyk2*, *Ly6c1*, and *Tbx21* have been replaced with *Tnfsf14*, *Cd28*, *Jak2*, and *Stat1*. Conclusions about all other genes—*Cxcr5*, *Tigit*, and *Ikzf4*—remain unchanged.

We note that while the significance levels of individual genes change, the conclusions drawn about the utility of the method are unaffected. We apologize for any confusion and provide a full description of the re-analysis and a list of exact changes to the text at GitHub (https://github.com/MarioniLab/RegressionBASiCS2017) and as a zenodo instance (https://doi.org/10.5281/zenodo.3382836). Additionally, we have provided a new diagnostic tool (BASiCS_diagPlot function) to guide the user and to ensure MCMC chains have indeed converged whenever the tool is applied. This new diagnostic tool is available in BASiCS 1.6.0 (https://doi.org/10.18129/B9.bioc.BASiCS) as part of the 3.9 Bioconductor release.

**Figure:**
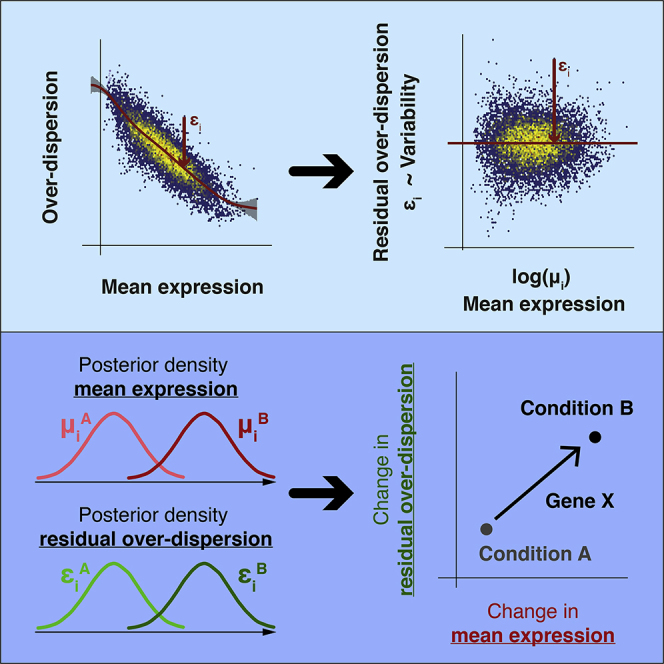
Graphical Abstract (corrected)

**Figure:**
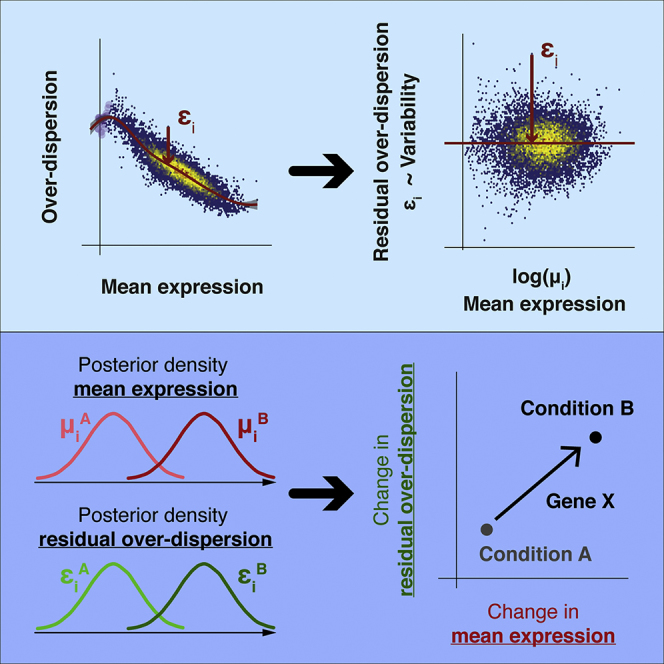
Graphical Abstract (original)

**Figure:**
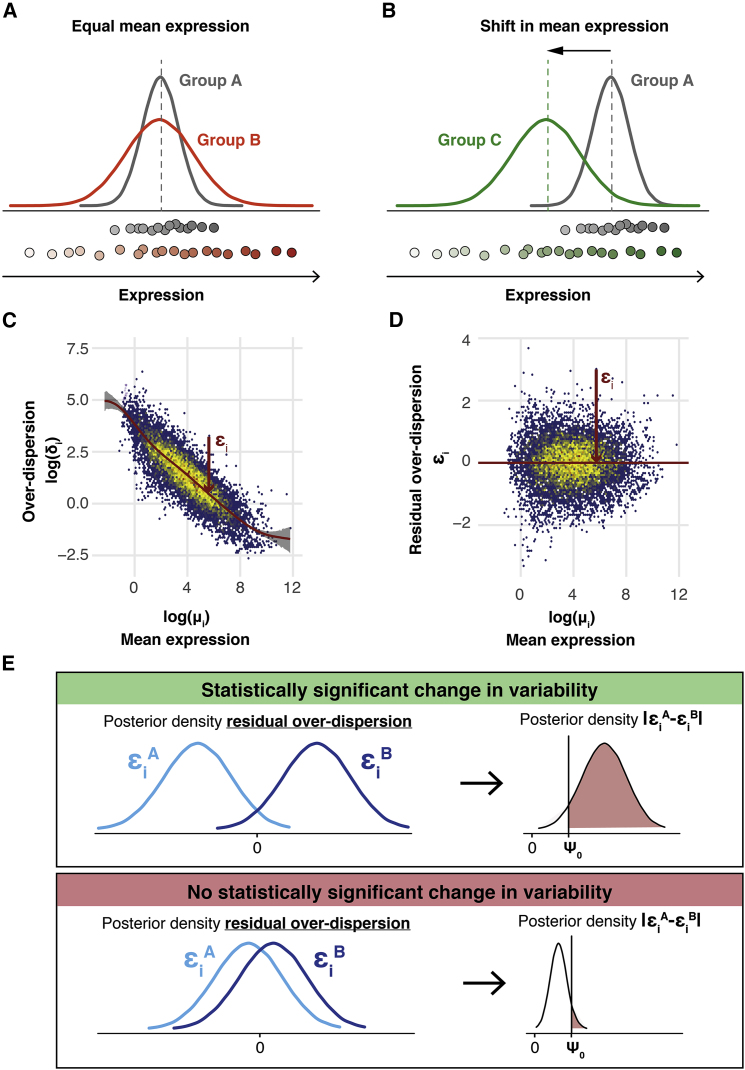
Figure 1. Avoiding the Mean Confounding Effect When Quantifying Expression Variability in scRNA-Seq Data (corrected)

**Figure:**
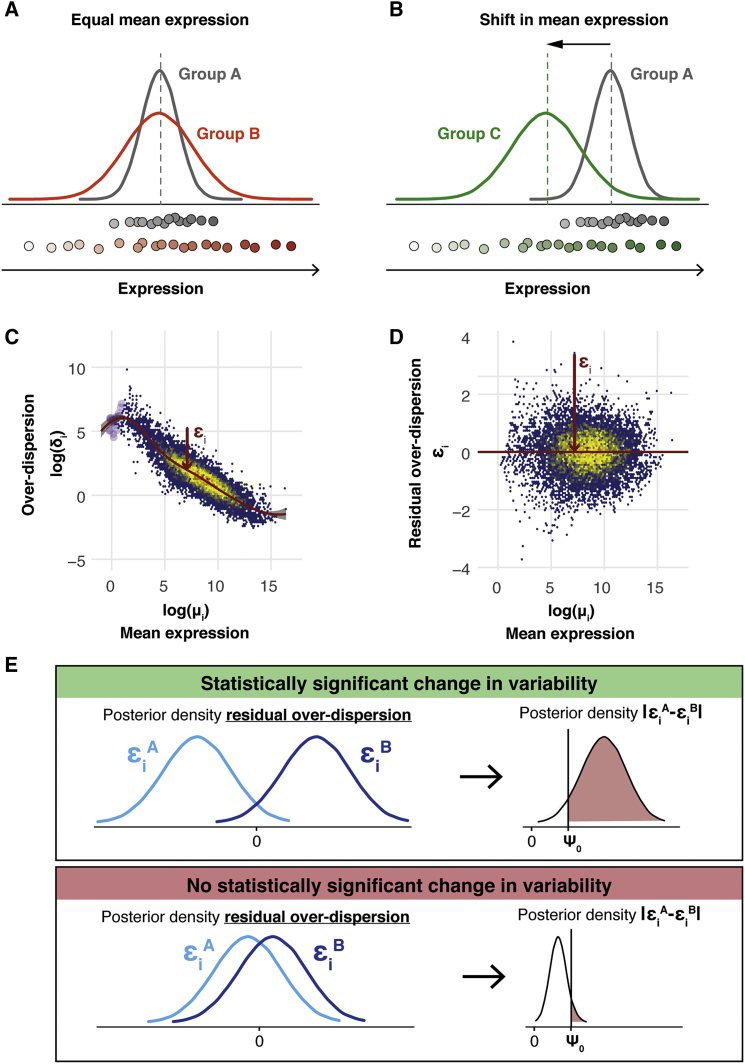
Figure 1. Avoiding the Mean Confounding Effect When Quantifying Expression Variability in scRNA-Seq Data (original)

**Figure:**
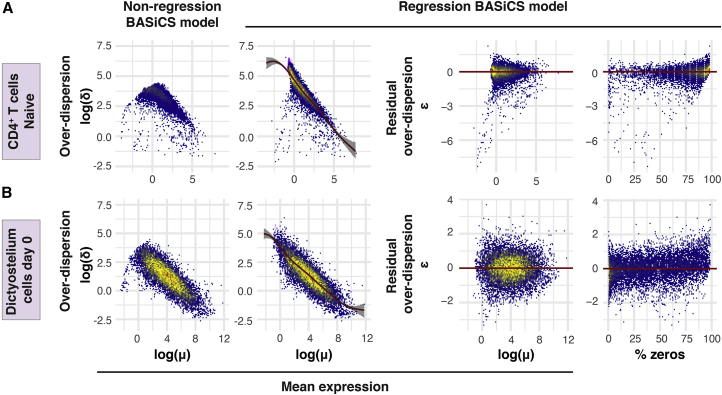
Figure 2. Parameter Estimation Using a Variety of scRNA-Seq Datasets (corrected)

**Figure:**
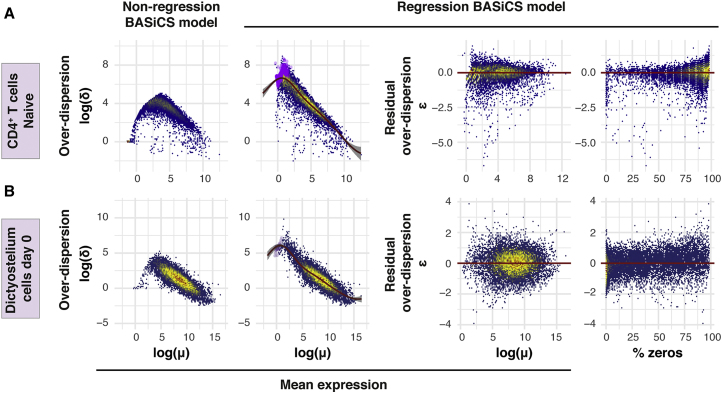
Figure 2. Parameter Estimation Using a Variety of scRNA-Seq Datasets (original)

**Figure:**
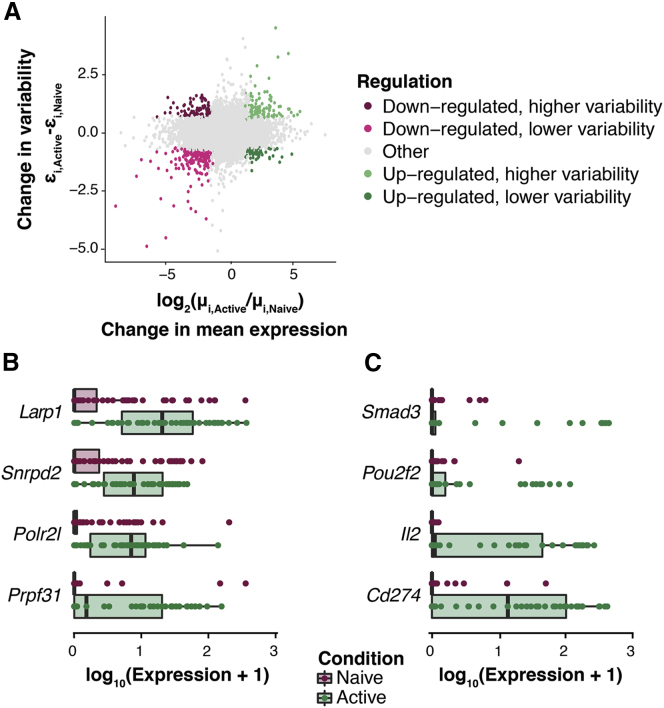
Figure 5. Changes in Expression Patterns during Early Immune Activation in CD4^+^ T Cells (corrected)

**Figure:**
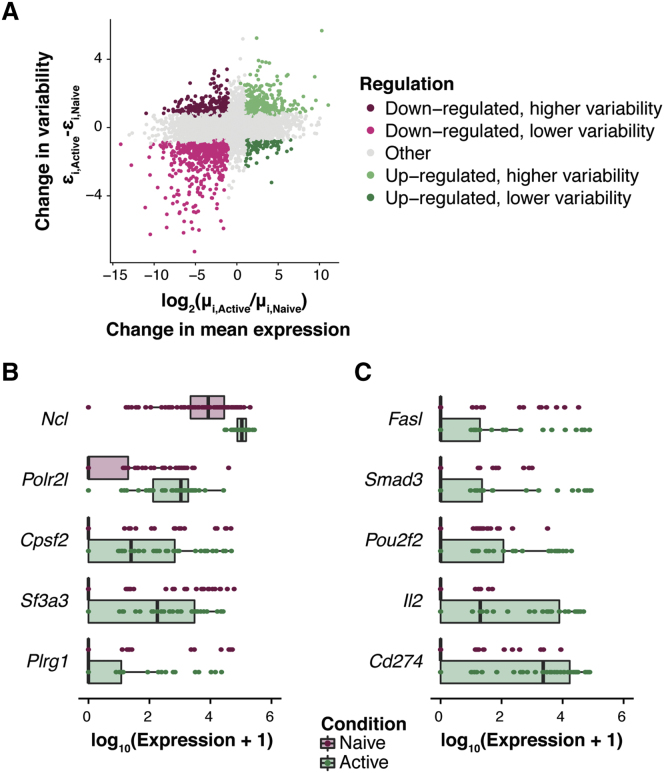
Figure 5. Changes in Expression Patterns during Early Immune Activation in CD4^+^ T Cells (original)

**Figure:**
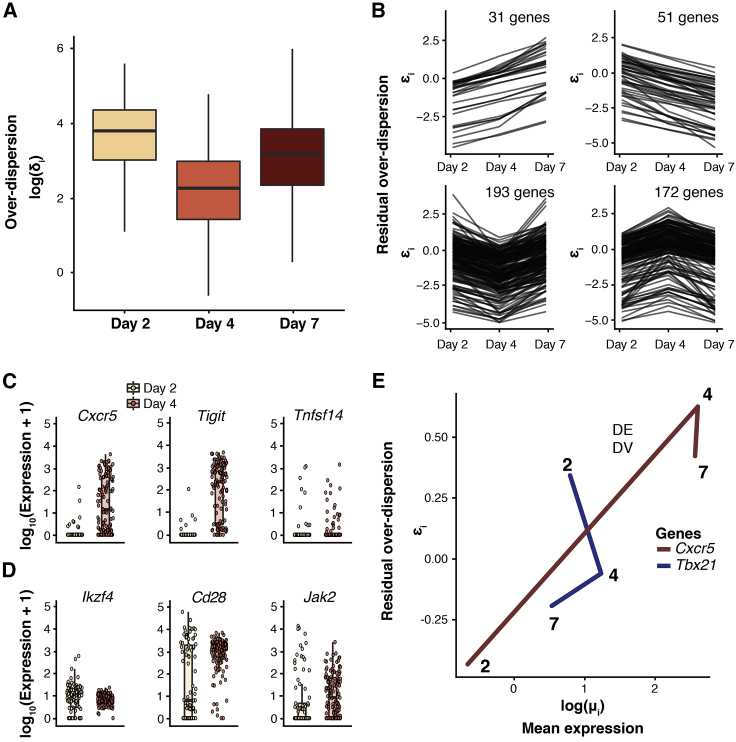
Figure 6. Dynamics of Expression Variability throughout CD4^+^ T Cell Differentiation (corrected)

**Figure:**
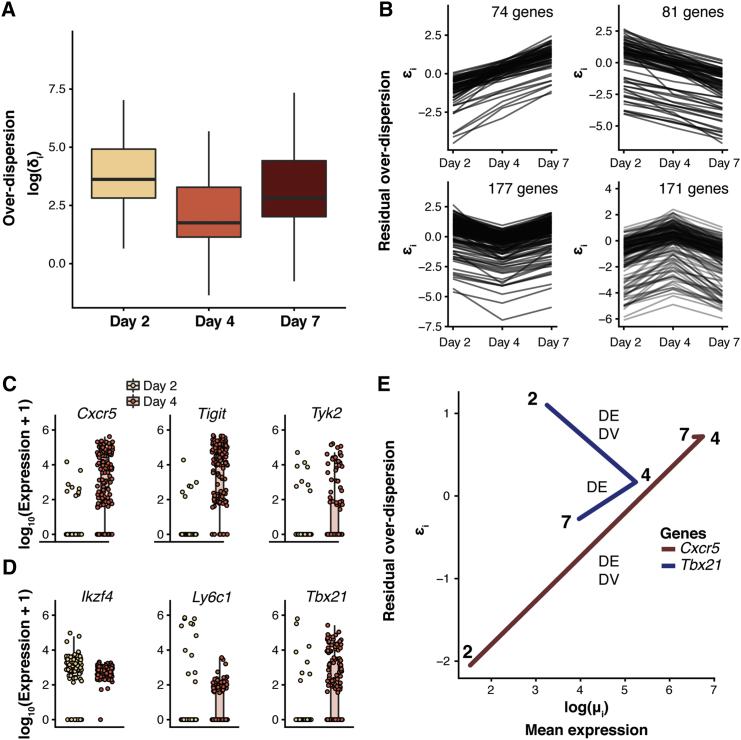
Figure 6. Dynamics of Expression Variability throughout CD4^+^ T Cell Differentiation (original)

**Figure:**
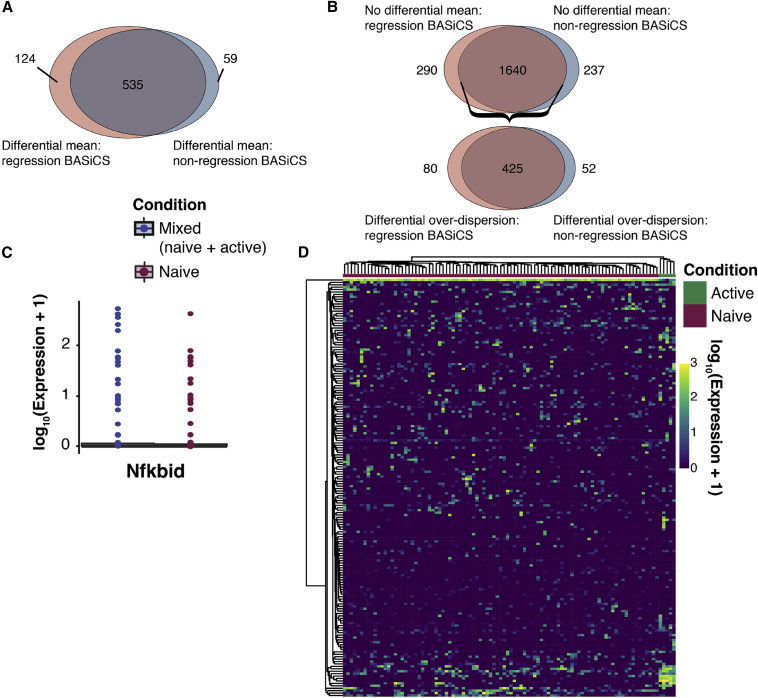
Figure S5 (corrected)

**Figure:**
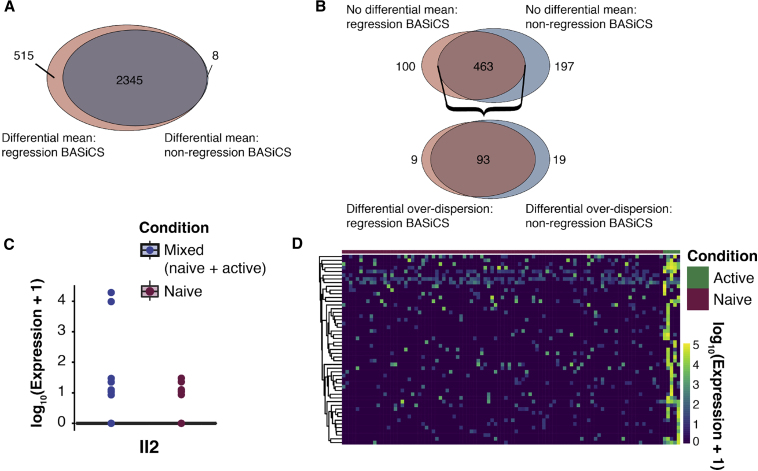
Figure S5 (original)

**Figure:**
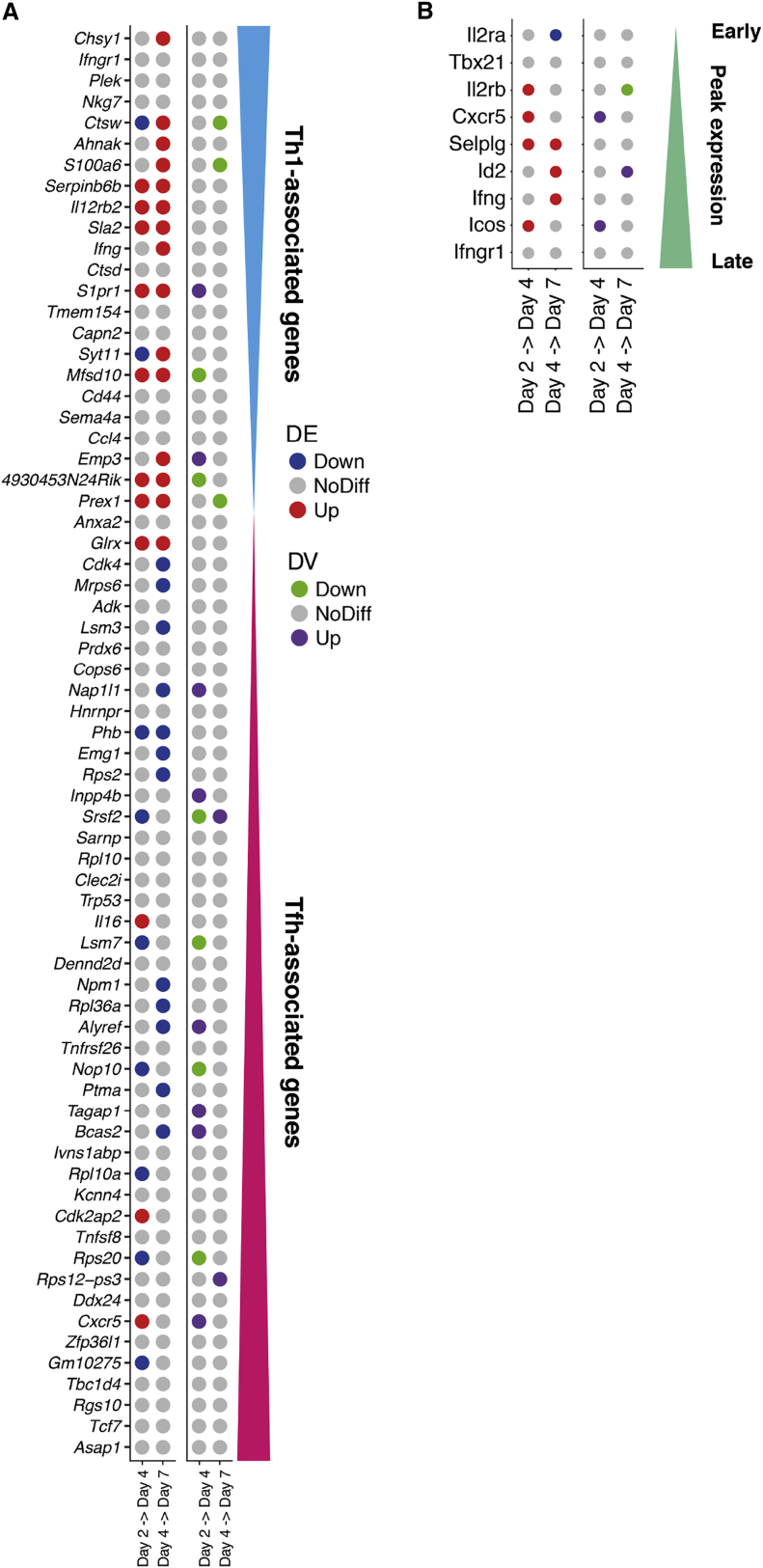
Figure S6 (corrected)

**Figure:**
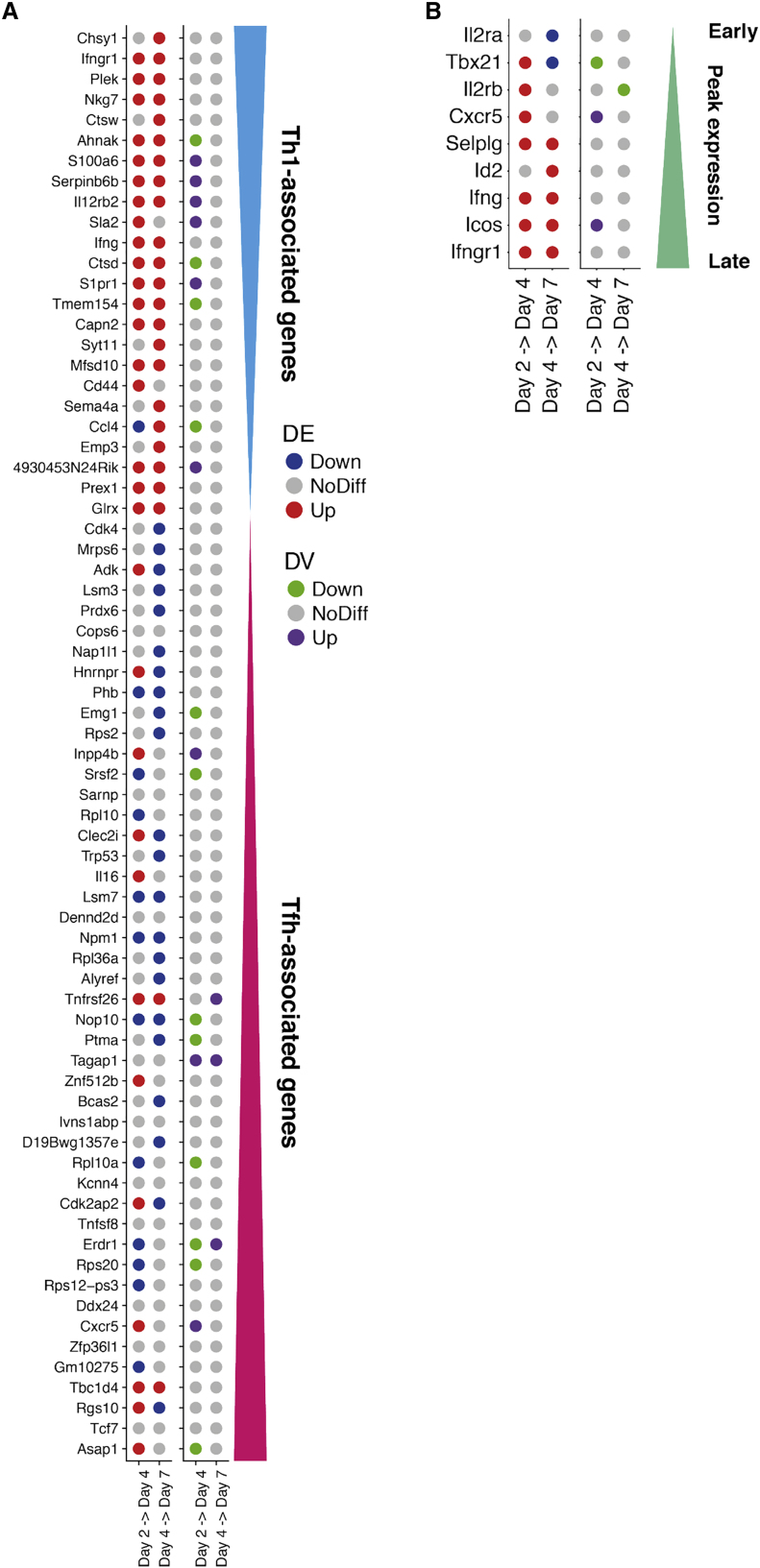
Figure S6 (original)
